# Institutions, people, knowledge: rebuilding the capacities of global health diplomacy

**DOI:** 10.1093/eurpub/ckag181

**Published:** 2026-10-09

**Authors:** Natia Skhvitaridze, Amiran Gamkrelidze

**Affiliations:** ^1^School of Health Sciences, Global and European Health Education and Study Institute, The University of Georgia, Tbilisi, Georgia; ^1^School of Health Sciences, Global and European Health Education and Study Institute, The University of Georgia, Tbilisi, Georgia

## Introduction

In 2007, the Oslo Ministerial Declaration identified health as ‘one of the most important, yet neglected, long-term foreign policy issues of our time’ [1]. Two decades later, that neglect is no longer benign. The January 2026 withdrawal of the USA from the World Health Organization (WHO), a widening pattern of donor retrenchment, and the contested implementation of the WHO Pandemic Agreement have placed multilateral health cooperation under its most severe stress in decades. Much of the ensuing debate has concentrated on institutions: budgets, treaties and governance reform. This focus is necessary but incomplete. Global health diplomacy rests on three interdependent capacities: institutions that negotiate and finance cooperation; people trained to work across the professional boundaries where diplomacy actually happens; and knowledge systems that determine whose evidence informs policy. Recent shocks have damaged all three simultaneously, and failures in one layer propagate to the others. Reform agendas that address institutions alone therefore risk rebuilding a structure on eroding foundations. This viewpoint examines each capacity in turn and draws out their intersections.

## The institutional layer: retrenchment and its spillovers

The institutional layer is the most visible casualty. Analyses of the USA withdrawal estimate a $600 million shortfall in the WHO’s 2025 budget, projected to reach $1.5 billion in 2026, with restructuring plans that would reduce staff by ∼28% and consolidate programme divisions from 10 to 4 [2]. The retreat also risks becoming contagious: Argentina announced a parallel withdrawal, several other governments signalled similar intentions, and France, Germany and the Netherlands reduced foreign assistance budgets amid competing defence priorities [2]. The America First Global Health Strategy, which replaces multilateral assistance with time-limited bilateral compacts, has been criticized for transferring fiscal responsibility to recipient countries faster than decision-making authority [3]. These shocks land on financing arrangements that were already fragile: official development assistance (ODA) from members of the Organisation for Economic Co-operation and Development (OECD) Development Assistance Committee averaged 0.34% of gross national income in 2024, half the long-standing 0.7% commitment [4], and although the World Bank Pandemic Fund had mobilized nearly $7 billion across 75 countries by early 2026, demand far exceeds supply [5]. The Pandemic Agreement, adopted in May 2025, is a genuine institutional innovation [6], but its annex on technology transfer and benefit-sharing remains unfinished, and compliance with the 2024 amendments to the International Health Regulations depends on trust and technical capacity that treaties cannot legislate into existence. Notably, modelling suggests that donor cuts may also be self-defeating, eroding the donors’ own health security and diplomatic influence [7].

## The human layer: workforce as infrastructure and as instrument

Institutions do not negotiate; people do. The Pandemic Agreement required years of negotiation among virologists, epidemiologists, global and public health specialists, economists, intellectual property lawyers and heads of state; no single profession could have produced it, and no single discipline can evaluate it [6]. Yet the training pipelines for health attachés, epidemiologists advising trade delegations and bioethicists supporting treaty negotiators rarely overlap, producing practitioners fluent in their discipline but ill-equipped for the interprofessional environments where real health diplomacy occurs.

The workforce is not only infrastructure for diplomacy; it can become an instrument of it. The dispute over Cuba’s medical missions illustrates the point. Cuba reportedly deploys >24 000 doctors across 56 countries [8], many in health systems with few alternatives. The US administration characterizes the programme as forced labour and has imposed visa restrictions on officials of participating states, prompting withdrawals by Guatemala and others, while several Caribbean governments maintain that their pandemic response would have been impossible without Cuban personnel [8]. The labour-rights concerns deserve scrutiny on their own terms; the narrower point here is that when the health workforce becomes geopolitical leverage, the immediate consequences fall on the populations that depend on it.

Strengthening this layer requires making interprofessional education a structural feature of health diplomacy training rather than an elective. Postgraduate and continuing professional development (CPD) programmes, such as those at the Geneva Graduate Institute, the Stockholm School of Economics-Karolinska Institute collaboration, Georgetown University and the University of Georgia, offer workable models that could be expanded and co-designed with practitioners from low- and middle-income countries (LMICs).

## The knowledge layer: whose evidence counts

The third capacity determines whose evidence reaches the first two. Article processing charges (APCs) at major open-access journals have risen sharply over the past decade, and higher APCs are associated with declining first authorship from LMICs, although the causal pathways are difficult to isolate [9]. A publishing model designed to democratize access to science has, in many settings, shifted the financial barrier from readers to authors. Declining health ODA compounds the effect: when researchers in LMICs cannot afford to publish, their findings are excluded from the evidence base on which global policy is built.

The three layers intersect here most visibly. The January 2025 US foreign aid freeze halted dozens of trials across Africa and Asia, including an HIV vaccine study spanning eight African countries whose regulatory approvals, trained staff and in-country vaccine supplies were rendered inactive within days [10]. A single institutional decision thus simultaneously destroyed research infrastructure, stranded trained professionals and truncated the evidence base: damage to all three capacities at once, with recovery times measured in years rather than budget cycles.

## Implications

Three implications follow, one per capacity. Institutionally, the Pandemic Agreement’s annex on technology transfer should be finalized with enforceable equity provisions rather than aspirational language, and donors falling short of the 0.7% ODA commitment could face structured accountability mechanisms within multilateral fora. For the workforce, global health diplomacy should become a required module in postgraduate and CPD curricula across health and policy disciplines, with certification pathways spanning epidemiology, negotiation, international law and health financing, co-created with practitioners from LMICs. For knowledge systems, journals and funders should adopt transparent, automatically applied APC waivers for authors from LMICs, report waiver uptake by country income group, support diamond open-access models and prioritize publication of null results and implementation research from resource-limited settings.

None of these reforms are sufficient alone; the framework’s central implication is that they are complements, not substitutes. Training more boundary-spanning professionals will achieve little if the institutions they would staff are defunded, and refinancing institutions will not correct policies built on a truncated evidence base.

## Conclusion

Viewing global health diplomacy as three interdependent capacities: institutions, people and knowledge, explains why the current reform debate, focused overwhelmingly on the first, is incomplete, and why shocks such as the US withdrawal cause damage that budget lines understate. The question is not whether health is political; it always has been. The question is whether the systems that train people and produce evidence will be rebuilt alongside the institutions that depend on them, before the next crisis makes the cost of neglect undeniable.

## Data Availability

No new data were generated or analysed in support of this Viewpoint. All sources drawn upon are publicly available and cited in the reference list.
